# Challenges to Design and Develop of DNA Aptamers for Protein Targets. II. Development of the Aptameric Affinity Ligands Specific to Human Plasma Coagulation Factor VIII Using SEC-SELEX

**Published:** 2017

**Authors:** Hossein Vahidi, Nastaran Nafissi-Varcheh, Bahram Kazemi, Reza Aboofazeli, Soraya Shahhosseini, Maryam Tabarzad

**Affiliations:** a *Department of Pharmaceutical Biotechnology, School of Pharmacy, Shahid Beheshti University of Medical Sciences, Tehran, Iran. *; b *Cellular and Molecular Biology Research Center, Shahid Beheshti University of Medical Sciences, Tehran, Iran.*; c *Biotechnology Department, School of Medicine, Shahid Beheshti University of Medical Sciences, Tehran, Iran.*; d *Department of Pharmaceutics, School of Pharmacy, Shahid Beheshti University of Medical Sciences, Tehran, Iran.*; e *Department of Pharmaceutical Chemistry, School of Pharmacy, Shahid Beheshti University of Medical Sciences, Tehran, Iran*; f *Protein Technology Research Center, Shahid Beheshti University of Medical Sciences, Tehran, Iran.*

**Keywords:** Aptamer, Protein, Coagulation Factor VIII, Affinity Ligands, Size Exclusion Chromatography, SELEX

## Abstract

Protein specific aptamers are highly applicable affinity ligands in different fields of research and clinical applications. They have been developed against various targets, in particular, bio-macromolecules such as proteins. Among human proteins, the coagulation factors are the most attractive targets for aptamer selection and their specific aptamers have valuable characteristics in therapeutic and analytical applications. In this study, a plasma derived coagulation factor VIII was considered as the protein target for DNA aptamer selection using size exclusion chromatography-SELEX. Potential aptameric oligonucleotides with high affinity and specificity were achieved during eight rounds of selection. Binding affinity constant of selected aptamer and aptameric enriched pool were in nanomolar range that was comparable to monoclonal antibodies. Further improvement studies can result in aptamers that are more promising as an industrial affinity ligand for the purification of anti-hemophilia factor from plasma source.

## Introduction

Proteins are a major group of biomolecules that play important roles in physiological and pathological processes in the living organisms, especially in human. They are the main component of many physiological events during different pathways. Introducing selective technologies like monoclonal antibodies and aptamers to the world of protein science have brought new insights into the basic knowledge and clinical applications. 

Aptamers are the single stranded oligonucleotides, which bind to their specific targets with high affinity and selectivity, according to their 3D structures. There are a broad range of targets from small molecules to large macromolecules, like proteins, and even whole cells or organisms (1). Nowadays, aptamers have been evaluated for numerous applications, as an alternative for the monoclonal antibodies. Aptamers have been extensively studied for the analytical applications such as affinity chromatography (2, 3). 

Selection of protein specific aptamers is performed according to the methods of «Systematic Evolution of Ligands by EXponential enrichment» (SELEX) and various related subtypes, in addition to the non-SELEX technologies (4). During SELEX, a random single stranded oligonucleotide library is incubated with target molecules and then, bound oligonucleotides are selected and separated from the non-bound oligos using different partitioning techniques, such as electrophoresis, chromatography, membrane filtration, and dialysis. Enriched pool of oligonucleotides, then, amplifies and enters to a new selection-partitioning-amplification cycle. At the end of SELEX process, when no more increase in the binding affinity was observed, the selected aptameric sequences are cloned and characterized (5). Size exclusion chromatography (SEC), as a partitioning technique, could result in the development of aptameric ligands in solution, without the target immobilization on solid supports. It would be valuable, especially in the selection process of protein specific aptamers, since the conformational alterations induced by surface attachment will be minimized. 

Clotting factor VIII (FVIII) is one of the non-enzymatic cofactor in the blood coagulation pathways that after activation, interacts with FIXa to trigger the factor X (FX). If there are defects in the FVIII gene on chromosome X, the patients suffer from a bleeding disorder called hemophilia A. By November 2012, more than two thousand various mutations in FVIII gene had been described that resulted in bleeding disease (6). Since 1960s, replacement therapy has been applied for emergency and prophylaxis treatment of hemophilia using FVIII concentrates. Currently, Coagulation FVIII products as the main hemophilia treatment is supplied from two sources, including extraction from human plasma or production by recombinant technology (7). 

Treatment with coagulation factor VIII is an expensive therapy, which cost approximately 100 000 US$ per year for one patient. Accordingly, numerous studies have focused on the improvement in the supply, safety and also, cost of the pharmaceutical factor VIII product by optimizing production, isolation, and purification process (8). Currently, there are various methods and affinity ligands for specific purification of factor VIII that monoclonal antibodies are the most established ones (9-12). Other affinity ligands applied for purification of this coagulation factor are heparin and "one" specific peptide ligand (13).

This study was aimed to select high affinity specific aptamers against coagulation factor VIII that could be considered as an affinity ligand candidate for protein purification or analysis. The SELEX technology with size exclusion chromatography (SEC) in partitioning step was used to develop specific aptameric ligands. Final aptamers could be considered as potential affinity ligands to develop an aptamer based affinity chromatography for FVIII purification from plasma source. 

## Material and Methods

*Materials*

PCR set including Taq DNA polymerase (5IU/μL), PCR 10x buffer (500 mM KCl and 200 mM Tris-HCl, pH 8.4), and MgCl_2_ solution (100 mM) were purchased from SinaClone (Iran). HPLC grade random single stranded DNA library, forward and reverse primers were synthesized by Metabion Company (Germany) at the concentration of 200 nM. TAE 10x buffer (1x buffer contains 40 mM Tris, 20 mM acetic acid and 1 mM EDTA) was obtained from Fermentas Company (Canada). 50-1500 bp DNA ladder and 6x loading dye (bromophenol blue and xylene cyanol) were prepared from GeneOne Company (Germany) and Vivantis Company (Malaysia), respectively. SafeStain dye and molecular grade agarose were obtained from SinaClone (Iran). Purified water was supplied from Direct-Q3 system (Millipore, USA). Sephadex superfine G75 was purchased from Pharmacia (Sweden). Culture media of LB broth and LB agar were supplied from Merck (Germany) and SOC medium from Sigma-Aldrich (USA). Plasmid extraction kit and DNA gel extraction kit was purchased from Bioneer (Korea). Plasma derived coagulation factor VIII (Haemoctin 500) was obtained from Biotest (UK). Factor VIII deficient plasma was supplied from Technoclone (Austria). 

**Table 1:** incubation conditions considered in SELEX process

**Rounds of SELEX**	**ssDNA pool conc.** **ng/µL**	**Protein FVIII conc.** **ng/µL**	**Incubation time**
1	45	100	16 h
2	40	100	16 h
3 negative selection	30	1 mL of FVIII deficient plasma	3 h
4	30	75	8 h
5	25	75	8 h
6	20	75	9 h
7 negative selection	10	1mL of FVIII deficient plasma	3 h
8	10	50	4 h

**Figure 1:**
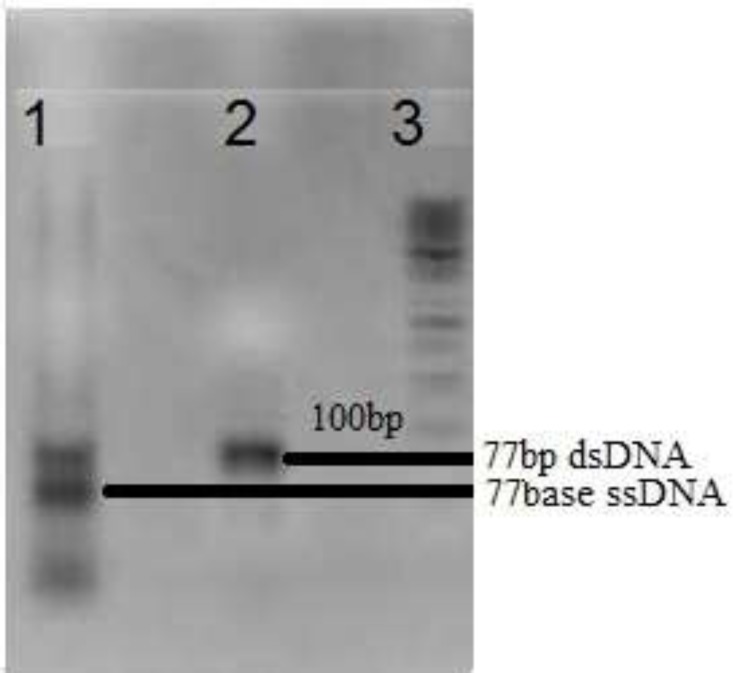
Gel electrophoresis of amplified DNA pool on 2% agarose.Lane 1: Asymmetric PCR amplification, Lane 2: PCR amplification and Lane 3:50bp DNA ladder

**Figure 2:**
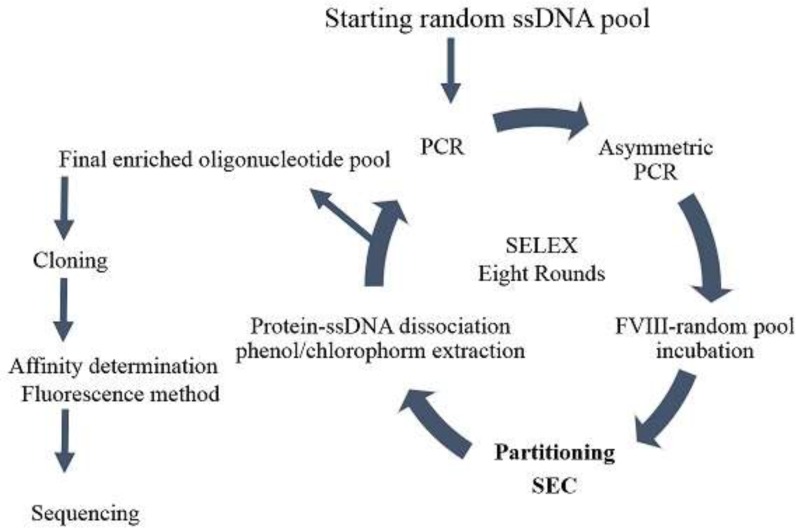
schematic presentation of the SELEX process utilized to develop FVIII specific aptamer. Eight rounds of selection was run to enrich the aptameric ssDNA pool. Counter selection using FVIII deficient plasma was run at 3rd and 7th round. Final enriched pool was clones and sequenced

**Figure 3:**
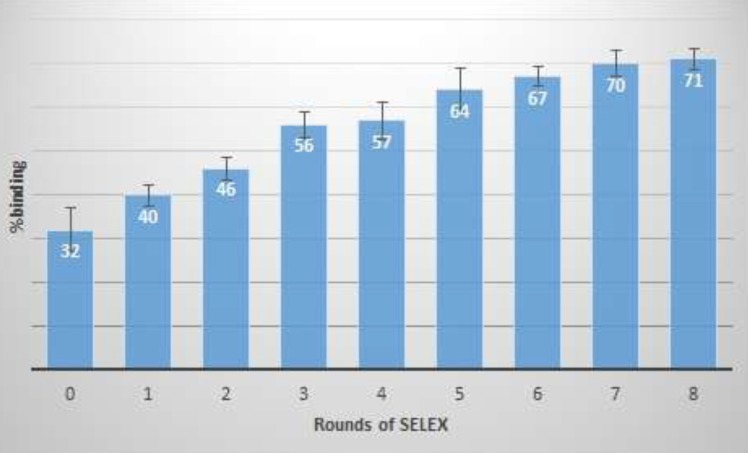
binding percentage of enriched ssDNA pool after different rounds of SELEX. The binding percentage was calculated using fluorescence absorbance of free oligonucleotides before and after target incubation

**Figure 4:**
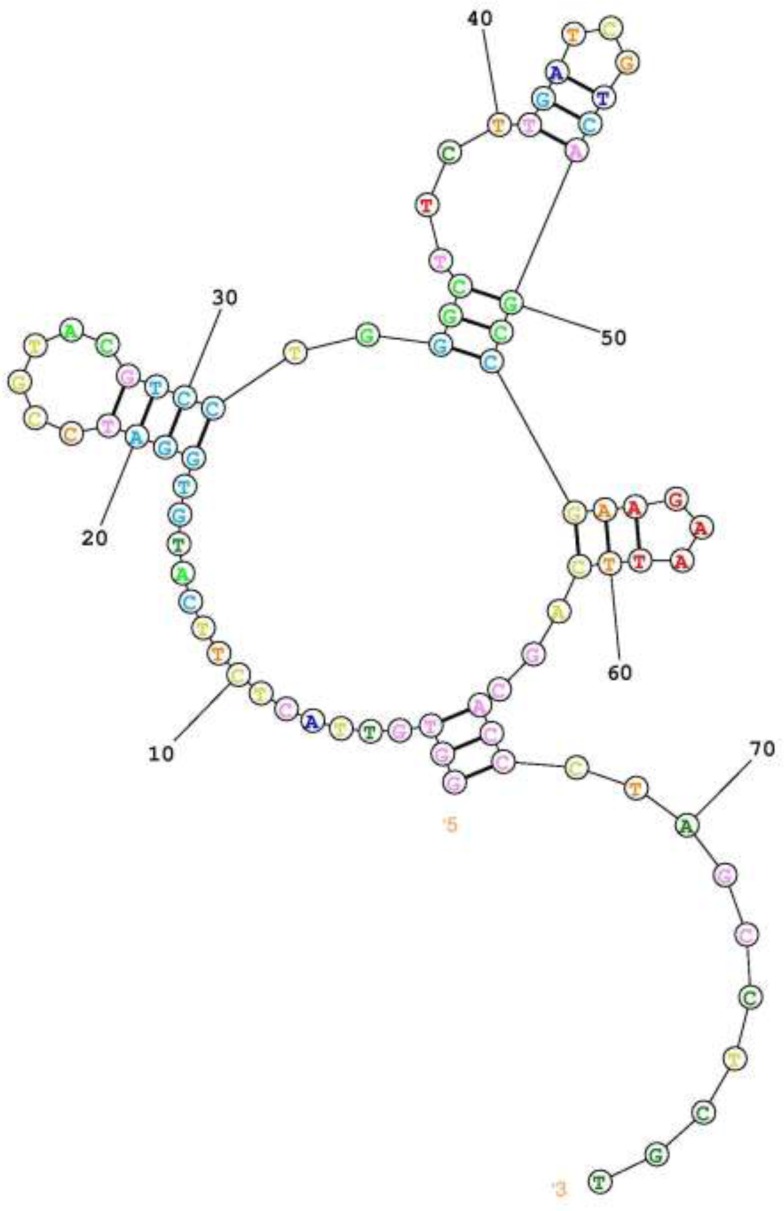
predicted secondary structure of the selected FVIII specific aptamer. Using online server of RNAStructure. Predict the lowest free energy structure

*Library design and amplification*

A DNA pligonucleotide of 30 nucleotides random DNA region flanked by two fixed regions of 23 and 24 nucleotides in the sequence of 5’-GGTGTTACTCTTCATGTGGATCCG (N30) AGAATTCAGCACCCTAGCCTCGT-3’ was designed and synthesized chemically (Metabion, Germany). Amplification of the random pool was run by PCR method using 24nts forward primer, 5’-GGTGTTACTCTTCATGTGGATCCG-3’ and 23nts reverse primer, 5’-ACGAGGCTAGGGTGCTGAATTCT-3’. Five nanograms of random DNA pool were used as starting template. PCR amplification program was a 2 min denaturation at 95 °C, by 30 repeats of thermal cycling included 95°C(30sec),64 °C (45 sec), and 72°C (15 s) and also final 5 min polymerization at 72°C. To prepare a single stranded random DNA library, amplified dsDNA pool was introduced directly to the asymmetric PCR including 35 cycles of amplification similar to the PCR protocol except the ratio of the forward to the reverse primer which kept at 1 to 10 (14).

*SELEX process*

Purified ssDNA oligonucleotide was thermally treated to form reproducible secondary structures. So, it was firstly heated at 95°C for 10 min; then cooled down immediately to 4°C, on ice for 5 min and finally kept at room temperature for additional 5 min to complete the formation of secondary structures. This 3D random ssDNA pool was incubated with Coagulation factor VIII solution in the binding buffer (Tris 40 mM, NaCl 117 mM, CaCl_2_ 5mM and MgCl_2_ 5 mM, pH = 7.4) for defined time at 4°C. Then, bound oligonucleotides were separated from the unbound ones using SEC. This partitioning step was run at 1*20 cm column of Sephadex G75 and flow rate of 

1 mL/min at 4 °C. Coagulation factor VIII and their bound oligonucleotides passed the column in void volume and collected in first 20 mL elution volume. The assay of the elution fractions for FVIII protein was performed by measuring the changes in 280 nm absorption using spectroscopy (ScanDrop, Analytik Jena, Germany). The collected samples were concentrated by Vivacon, cutoff 50 KDa (Sartorius, Germany) during 2 min of centrifugation at 4 °C and 8000 rpm. Concentrated solution in less than 0.5 mL was collected and after phenol/chloroform extraction, the aqueous phase was applied to the PCR and Asymmetric PCR amplification process. After that, this new enriched ssDNA pool was purified using agarose gel electrophoresis and applied for the next round of selection. 

*Counter SELEX*

Coagulation factor VIII deficient plasma was re-suspended by 2 mL binding buffer. In 3^rd^ and 7th round of SELEX, the enriched pool from previous round of selection was amplified, purified; and incubated to 1 mL of deficient plasma for 30 min in 4 °C. Incubation mixture was passed through 0.45 µM nitrocellulose membrane filter (Sartorius, Germany). Then, the filtrate was considered for the next round of selection. 

*Cloning and Sequencing*

The final enriched aptameric pool was cloned using InsTAclone PCR Cloning Kit (Fermentas, USA). White recombinant clones were selected and plasmids were extracted. Purified plasmids were sequenced by a pair of universal primers. Then, sequences were analyzed by BlastN software of NCBI and aptamer sequences were de-convoluted according to the specific forward and reverse primers. 

*Affinity determination*

Dissociation constant (Kd) of enriched libraries and individual aptamers were determined by fluorescence method. Enriched aptameric pool was amplified by PCR followed asymmetric PCR in the presence of the fluorinated reverse primer (Fluorescein) using Pfu DNA polymerase. Then, labeled oligonucleotide pool was diluted to a set of twelve concentration (from 0.5 to 700 nM) and incubated with 20 ng coagulation factor VIII (equal to 700 pM). All test samples were run triplicated. A similar set of 12 different concentrations of labeled oligonucleotides was prepared as standards to draw calibration curve for fluorimetry. 

Nitrocellulose filtration was applied to separate bound from unbound oligonucleotides. Filtration run under the vacuum and the filter was washed three times with binding buffer to remove unbound oligonucleotides completely. Then, the fluorescence absorption of filtrates was measured by fluorescence spectroscopy (F2500 fluorimeter, Hitachi, Japan). Excitation and emission wavelength were set at 492 nm and 521 nm, respectively. Difference between standards and samples fluorescence absorption was calculated as the quantity identifier of the bound oligonucleotides. The results were analyzed by GraphPad Prism 5 and the binding constant (Kd) was calculated according to the method of non-linear regression and binding-saturation, one site-total. 

## Results and Discussion

*Preparation and amplification of Random DNA library*

A random ssDNA pool was designed by 30nts central random region which provide efficient diversity of 10^13^-10^15 ^various 3D structures of oligonucleotides. Fixed flanking region composed of two ~ 20 nts that served as primer binding region during PCR amplifications. For the first round of selection, the designed 77 nts random DNA oligonucleotide was amplified with PCR and then, asymmetric PCR to develop a random single stranded DNA library. For the following selection rounds, recovered DNA library was first amplified by PCR and then converted to ssDNA pool omit "of DNA" by Asymmetric PCR (Figure 1). As showed in Figure 1, in the molar ratio equal to 10 of reverse to forward primers, a significant increase in the amount of ssDNA compared to that of dsDNA can be seen after 35 cycles of amplification. Then, ssDNA was purified and applied to the next round of selection. Concentration of the recovered DNA solution (regarding 260 nm absorption) was decreased as the selection round increased. Accordingly, for the 8^th^ round of SELEX, the value after 35 cycles of amplification was less than 0.01 ng/µL. 

In aptamer technology, 3D structures of the single stranded oligonucleotides play an important role in binding to the targets. Accordingly, reproducible induction of the 3D structures in random ssDNA pool was essential at the beginning of each round. Applying the same thermal treatment before starting each round of selection process supports the reproducible formation of 3D structure for individual sequences during different rounds of selection. 

*In-vitro selection of target specific oligonucleotide ligands*

In vitro selection of FVIII-specific DNA aptamer was run using SELEX method based on size exclusion chromatography in partitioning steps (Figure 2). Starting random oligonucleotide libraries for in-vitro aptamer selection have been usually designed to cover bout 10^13-15 ^different sequences. According to the published studies, a random region of about 30 nts can gratify the desired diversity (15-17). Coagulation factor VIII is a large glycoprotein of 2332 amino acids with multi domain structure and has an important role in intrinsic blood coagulation cascade (18, 19). The molecular mass of the 77 nts ssDNA oligonucleotide was around 20 KDa, while the molecular mass of plasma deriving coagulation FVIII was about 300 KDa. Accordingly, Sephadex G75 with the fractionation range of 3-70 KDa was selected as fixed column for SEC. Therefore, the protein of FVIII and bound oligonucleotides eluted in void volume, whereas, free oligonucleotides enter the pores of column and take a longer time to pass through the system. The FVIII protein is a large glycoprotein with high sensitivity to environmental conditions (20) that result in difficulties to arrange appropriate condition for selection process. To get maximum availability of protein epitope, it would be better to conduct aptamer selection by a freely soluble form, not immobilized protein. Accordingly, in this study, the protein-oligonucleotide library incubation step was performed in free solution and the bound and unbound oligonucleotides were separated by size exclusion chromatography according to the size differences (21). By this method, the incubation and the partitioning steps were run without solid support protein attachment. Keeping protein in solution during aptamer selection helps to conserve the target protein physiological conformation.

The concentrations of recovered ssDNA from each round of SELEX, after electrophoresis and gel extraction, were decreased as the number of SELEX cycle increased. Two rounds of negative selection (counter SELEX) were run to eliminate cross binding of selected oligonucleotides to the other plasma protein components. During negative selection, the enriched ssDNA pool from previous round was incubated with coagulation FVIII deficient plasma, then, followed by nitrocellulose membrane filtration. In order to stringent the selection condition, concentration of the protein solution and the incubation time were set to decrease. Consequently, the affinity of selected aptamers increased. Total eight rounds of selection (Table 1) were run and there was no significant difference between binding percentages in the last two consecutive selection rounds.

After eight rounds of SELEX, enriched ssDNA pool with high affinity to FVIII (Kd = 0.5 nM) was cloned and sequenced to reveal the aptameric sequences. As the enriched ssDNA pool contains same length different sequences, it is necessary to clone it or recently, run the next generation sequencing methods (22) to characterize aptameric sequences, individually. 

*Affinity determination of enriched pool*

At the end of the each round, a part of the ssDNA pool of selected oligonucleotides was considered for affinity measurement by the fluorescence method. Differences between fluorescence signals of free oligonucleotides, before and after the FVIII incubation, were measured and the percentage of binding was calculated as presented in Figure 3. 

As increasing in binding percentage was less than one between rounds of 7th and 8th, therefore, SELEX process was stopped and the Kd of the last enriched aptameric pool was determined. Binding constant (Kd) of the enriched aptameric pools at 4th and 8th round were 30 and 0.5 nM, respectively. As the enriched ssDNA pool affinity was increased by 60 times during the last four rounds, the SELEX process could be stopped at the 8^th^ round. Then, the final enriched ssDNA pool was cloned and sequenced. The calculated Kd of the enriched aptameric pool was comparable to the Kd of the specific monoclonal antibodies for coagulation FVIII. Cross-affinity of aptameric ligand to the Factor VIII deficient plasma was evaluated, similarly. The result showed no differences between fluorescence signal of free oligonucleotides before and after target incubation.

Binding constant (Kd) of the final selected aptamer was measured considerably greater than the Kd of the final enriched pool. This could indicate that the truncated aptameric sequences might have higher binding affinity or the sum of affinities of different aptamer sequences might result in the higher affinity of whole final enriched pool compared to the selected aptamer sequence.

*Aptamer sequencing*

Enriched pool of 8^th^ round was cloned. Among 10 clones that were selected and sequenced, one of the clones presented full length sequence. The others are truncated forms of aptameric oligonucleotides. This sequences was5’-GGTGTTACTCTTCATGTGGATCCGTACGTCCTGGGCTTCTTGATCGTCAGCCGAAGAATTCAGCACCCTAGCCTCGT-3’ and the dissociation constant of this aptameric sequence was calculated 925 ± 11.6 nM.

*Secondary structure prediction*

Secondary structure of the final aptameric ligand was predicted by online server of RNA Structure which indicate a central loop with three branches of stem loop structure (Figure 4). This arrangement may result in one rigid scaffold as the aptamer binding motif in the unstructured part. According to this prediction, the aptamer sequence can be further optimized by point mutation in unstructured part of binding motifs.

## Conclusion

Generally, human coagulation factor VIII is one of the plasma derived valuable therapeutic proteins, administered in bleeding disorders and covers a remarkable market worldwide. Improving FVIII production platform during plasma fractionation could reduce the cost of production and therefore, reduce final pharmaceutical product price. Presently, FVIII purification during plasma fractionation is performed by various chromatographic methods such as affinity chromatography and ion exchange. Specific ligands of affinity chromatography include a range from small molecules like heparin to large proteins, such as monoclonal antibodies. As a fact, a good affinity ligand for protein purification in industrial scale have to be small in size, stable with optimum affinity to bind and dissociate target in reproducible logical conditions, without adverse effect on target protein. It is also important that the ligand residue, leaked in the final product, will not affect the product safety and efficacy. Aptamers as single stranded oligonucleotides could cover the preferred properties of a high affinity ligand in protein purification. 

Currently, there are different products of anti-hemophilic factor in market purified by monoclonal antibody based affinity chromatography; e.g. Monoclate-P^® ^purified using a monoclonal antibody to vWF and Hemofil M^®^ purified by a murine monoclonal antibody to Factor VIII: C. Additionally, some companies such as Acris Antibodies, Abcam, Thermofisher and AbD serotec^®^ develop various types of human FVIII specific monoclonal antibodies for analytical applications. As a result, development of high affinity specific aptamers will be a promising alternative affinity ligands for most of analytical and industrial applications. 

In this study, coagulation FVIII-specific aptameric ligand was developed as an affinity ligand which could be applied for the protein affinity purification. Post SELEX modification of the selected aptamer could improve the ligand characteristics and will be investigated in the future studies to achieve a ligand with ideal stability, low toxicity, high affinity, and selectivity for protein purification or analysis. 
